# (2,2′-Bipyridine-κ^2^ *N*,*N*′)tetra­bromidoplatinum(IV)

**DOI:** 10.1107/S1600536810034793

**Published:** 2010-09-04

**Authors:** Kwang Ha

**Affiliations:** a School of Applied Chemical Engineering, The Research Institute of Catalysis, Chonnam National University, Gwangju 500-757, Republic of Korea

## Abstract

In the title complex, [PtBr_4_(C_10_H_8_N_2_)], the Pt^IV^ ion has a slightly distorted octa­hedral coordination defined by two N atoms of the chelating 2,2′-bipyridine ligand and four bromide ions. As a result of the different *trans* effects of the N and Br atoms, the Pt—Br bonds *trans* to the N atom are slightly shorter than those to mutually *trans* Br atoms. In the crystal structure, the mol­ecules are arranged in a V-shaped packing pattern along the *b* axis and stacked in columns along the *a* axis. In the columns, several inter­molecular π–π inter­actions between the pyridine rings are present. The shortest ring centroid–centroid distance is 3.921 (6) Å, with a dihedral angle of 1.6 (5)° between the ring planes. The complexes are connected by C—H⋯Br hydrogen bonds, forming chains along the *b* axis.

## Related literature

For the crystal structure of [PtCl_4_(bipy)] (bipy = 2,2′-bipyridine), see: Hambley (1986 ▶).
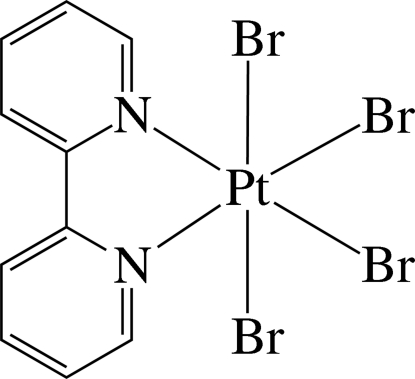


## Experimental

### 

#### Crystal data

[PtBr_4_(C_10_H_8_N_2_)]
*M* *_r_* = 670.91
Monoclinic, 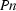

*a* = 8.3146 (7) Å
*b* = 6.9010 (5) Å
*c* = 12.5873 (10) Å
β = 102.940 (2)°
*V* = 703.91 (10) Å^3^
*Z* = 2
Mo *K*α radiation
μ = 21.30 mm^−1^
*T* = 200 K
0.25 × 0.12 × 0.08 mm

#### Data collection

Bruker SMART 1000 CCD diffractometer
Absorption correction: multi-scan (*SADABS*; Bruker, 2000 ▶) *T*_min_ = 0.462, *T*_max_ = 1.000
4282 measured reflections
2257 independent reflections
2128 reflections with *I* > 2σ(*I*)
*R*_int_ = 0.033

#### Refinement

*R*[*F* ^2^ > 2σ(*F* ^2^)] = 0.027
*wR*(*F* ^2^) = 0.053
*S* = 0.98
2257 reflections
154 parameters
2 restraints
H-atom parameters constrained
Δρ_max_ = 1.69 e Å^−3^
Δρ_min_ = −1.66 e Å^−3^
Absolute structure: Flack (1983 ▶), 714 Friedel pairs
Flack parameter: −0.004 (14)

### 

Data collection: *SMART* (Bruker, 2000 ▶); cell refinement: *SAINT* (Bruker, 2000 ▶); data reduction: *SAINT*; program(s) used to solve structure: *SHELXS97* (Sheldrick, 2008 ▶); program(s) used to refine structure: *SHELXL97* (Sheldrick, 2008 ▶); molecular graphics: *ORTEP-3* (Farrugia, 1997 ▶) and *PLATON* (Spek, 2009 ▶); software used to prepare material for publication: *SHELXL97*.

## Supplementary Material

Crystal structure: contains datablocks global, I. DOI: 10.1107/S1600536810034793/fj2332sup1.cif

Structure factors: contains datablocks I. DOI: 10.1107/S1600536810034793/fj2332Isup2.hkl

Additional supplementary materials:  crystallographic information; 3D view; checkCIF report

## Figures and Tables

**Table 1:** Selected bond lengths (Å)

Pt1—N2	2.046 (7)
Pt1—N1	2.048 (7)
Pt1—Br1	2.4412 (10)
Pt1—Br2	2.4442 (10)
Pt1—Br4	2.4595 (11)
Pt1—Br3	2.4756 (11)

**Table 2:** Hydrogen-bond geometry (Å, °)

*D*—H⋯*A*	*D*—H	H⋯*A*	*D*⋯*A*	*D*—H⋯*A*
C1—H1⋯Br2	0.95	2.73	3.366 (9)	125
C3—H3⋯Br1^i^	0.95	2.89	3.734 (10)	149
C10—H10⋯Br1	0.95	2.70	3.335 (9)	125

Symmetry code: (i) 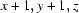.

**Fig. 1.**
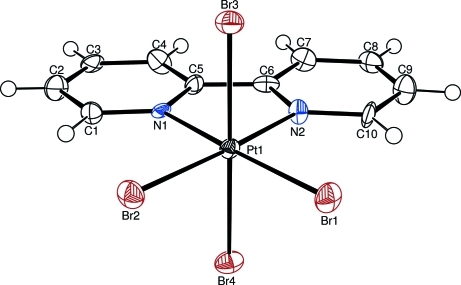
The structure of the title complex, with displacement ellipsoids drawn at the 50% probability level for non-H atoms; H atoms are shown as small circles of arbitrary radius.

**Fig. 2.**
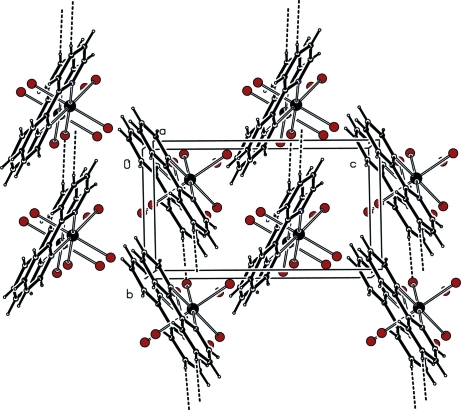
View of the unit-cell contents of the title complex. Intermolecular hydrogen-bond interactions are drawn with dashed lines.

## supplementary crystallographic information

### Comment

The title complex, [PtBr_4_(bipy)], is isomorphous with the chloro analogue [PtCl_4_(bipy)] (Hambley, 1986). The central Pt(IV) ion has a slightly distorted octahedral coordination defined by two N atoms of the chelating 2,2'-bipyridine ligand and four bromide ions (Fig. 1). The main contribution to the distortion of octahedron is the tight N1—Pt1—N2 chelate angle (80.6 (3)°), which results in non-linear *trans* axes (<Br1—Pt1—N1 = 176.0 (2)°, <Br2—Pt1—N2 = 176.0 (2)° and <Br3—Pt1—Br4 = 177.93 (4)°). As a result of the different *trans* effects of the N and Br atoms, the Pt—Br bonds *trans* to the N atom (2.4412 (10) Å and 2.4442 (10) Å) are slightly shorter than bond lengths to mutually *trans* Br atoms (2.4595 (11) Å and 2.4756 (11) Å) (Table 1). In the crystal structure, the complex molecules are arranged in a V-shaped packing pattern along the *b* axis and stacked in columns along the *a* axis (Fig. 2). In the columns, several intermolecular π-π interactions between the pyridine rings are present, with a shortest ring centroid-centroid distance of 3.921 (6) Å, and the dihedral angle between the ring planes is 1.6 (5)°. Moreover, there are intra- and intermolecular hydrogen bonds between the C and Br atoms with d(C···Br) = 3.335 (9) Å–3.734 (10) Å (Table 2). The complexes are connected by the C—H···Br hydrogen bonds, forming one-dimensional chains along the *b* axis.

### Experimental

To a solution of K_2_PtBr_6_ (0.1003 g, 0.133 mmol) in H_2_O (10 ml) was added 2,2'-bipyridine (0.0210 g, 0.134 mmol), and the mixture was refluxed for 3 h. The formed precipitate was separated by filtration, washed with water, and dried at 50 °C, to give an orange-yellow powder (0.0705 g). Crystals suitable for X-ray analysis were obtained by slow evaporation from an *N,N*-dimethylformamide solution at 50 °C.

### Refinement

H atoms were positioned geometrically and allowed to ride on their respective parent atoms [C—H = 0.95 Å and *U*_iso_(H) = 1.2*U*_eq_(C)]. The highest peak (1.69 e Å^-3^) and the deepest hole (-1.66 e Å^-3^) in the difference Fourier map are located 0.96 and 0.96 Å from the Pt1 atom, respectively.

### Crystal data

**Table:**

[PtBr_4_(C_10_H_8_N_2_)]	*F*(000) = 600
*M**_r_* = 670.91	*D*_x_ = 3.165 Mg m^−^^3^
Monoclinic, *P**n*	Mo *K*α radiation, λ = 0.71073 Å
Hall symbol: P -2yac	Cell parameters from 3381 reflections
*a* = 8.3146 (7) Å	θ = 2.7–27.0°
*b* = 6.9010 (5) Å	µ = 21.30 mm^−^^1^
*c* = 12.5873 (10) Å	*T* = 200 K
β = 102.940 (2)°	Block, orange
*V* = 703.91 (10) Å^3^	0.25 × 0.12 × 0.08 mm
*Z* = 2	

### Data collection

**Table:**

Bruker SMART 1000 CCD diffractometer	2257 independent reflections
Radiation source: fine-focus sealed tube	2128 reflections with *I* > 2σ(*I*)
graphite	*R*_int_ = 0.033
φ and ω scans	θ_max_ = 27.0°, θ_min_ = 3.0°
Absorption correction: multi-scan (*SADABS*; Bruker, 2000)	*h* = −8→10
*T*_min_ = 0.462, *T*_max_ = 1.000	*k* = −8→8
4282 measured reflections	*l* = −16→15

### Refinement

**Table:**

Refinement on *F*^2^	Secondary atom site location: difference Fourier map
Least-squares matrix: full	Hydrogen site location: inferred from neighbouring sites
*R*[*F*^2^ > 2σ(*F*^2^)] = 0.027	H-atom parameters constrained
*wR*(*F*^2^) = 0.053	*w* = 1/[σ^2^(*F*_o_^2^) + (0.0057*P*)^2^] where *P* = (*F*_o_^2^ + 2*F*_c_^2^)/3
*S* = 0.98	(Δ/σ)_max_ < 0.001
2257 reflections	Δρ_max_ = 1.69 e Å^−^^3^
154 parameters	Δρ_min_ = −1.66 e Å^−^^3^
2 restraints	Absolute structure: Flack (1983), 714 Friedel pairs
Primary atom site location: structure-invariant direct methods	Flack parameter: −0.004 (14)

### Special details

**Table:**

Geometry. All e.s.d.'s (except the e.s.d. in the dihedral angle between two l.s. planes) are estimated using the full covariance matrix. The cell e.s.d.'s are taken into account individually in the estimation of e.s.d.'s in distances, angles and torsion angles; correlations between e.s.d.'s in cell parameters are only used when they are defined by crystal symmetry. An approximate (isotropic) treatment of cell e.s.d.'s is used for estimating e.s.d.'s involving l.s. planes.
Refinement. Refinement of *F*^2^ against ALL reflections. The weighted *R*-factor *wR* and goodness of fit *S* are based on *F*^2^, conventional *R*-factors *R* are based on *F*, with *F* set to zero for negative *F*^2^. The threshold expression of *F*^2^ > σ(*F*^2^) is used only for calculating *R*-factors(gt) *etc*. and is not relevant to the choice of reflections for refinement. *R*-factors based on *F*^2^ are statistically about twice as large as those based on *F*, and *R*- factors based on ALL data will be even larger.

### Fractional atomic coordinates and isotropic or equivalent isotropic displacement parameters (Å^2^)

**Table:**

	*x*	*y*	*z*	*U*_iso_*/*U*_eq_	
Pt1	0.05995 (4)	0.30254 (5)	0.15692 (3)	0.01371 (9)	
Br1	−0.17951 (12)	0.09180 (15)	0.14380 (8)	0.0242 (2)	
Br2	−0.05252 (13)	0.51205 (15)	0.27909 (8)	0.0237 (2)	
Br3	0.21931 (13)	0.12002 (14)	0.31519 (8)	0.0232 (2)	
Br4	−0.08910 (13)	0.48563 (15)	−0.00258 (8)	0.0239 (2)	
N1	0.2650 (9)	0.4685 (11)	0.1594 (6)	0.0155 (18)	
N2	0.1677 (10)	0.1400 (11)	0.0559 (6)	0.0159 (18)	
C1	0.3042 (12)	0.6354 (13)	0.2127 (8)	0.018 (2)	
H1	0.2326	0.6863	0.2550	0.021*	
C2	0.4468 (14)	0.7363 (14)	0.2081 (9)	0.028 (3)	
H2	0.4746	0.8531	0.2479	0.034*	
C3	0.5471 (16)	0.6627 (13)	0.1441 (11)	0.024 (2)	
H3	0.6445	0.7304	0.1389	0.029*	
C4	0.5072 (13)	0.4909 (15)	0.0873 (9)	0.024 (2)	
H4	0.5768	0.4401	0.0436	0.029*	
C5	0.3659 (11)	0.3954 (14)	0.0951 (8)	0.018 (2)	
C6	0.3148 (12)	0.2147 (15)	0.0401 (8)	0.019 (2)	
C7	0.4026 (14)	0.1139 (14)	−0.0252 (8)	0.024 (2)	
H7	0.5045	0.1630	−0.0360	0.029*	
C8	0.3388 (13)	−0.0589 (14)	−0.0740 (8)	0.023 (2)	
H8	0.3975	−0.1291	−0.1182	0.027*	
C9	0.1902 (13)	−0.1282 (15)	−0.0583 (8)	0.027 (2)	
H9	0.1453	−0.2448	−0.0928	0.032*	
C10	0.1068 (12)	−0.0280 (13)	0.0075 (8)	0.021 (2)	
H10	0.0055	−0.0774	0.0190	0.025*	

### Atomic displacement parameters (Å^2^)

**Table:**

	*U*^11^	*U*^22^	*U*^33^	*U*^12^	*U*^13^	*U*^23^
Pt1	0.01098 (16)	0.01521 (16)	0.01523 (16)	−0.00154 (17)	0.00355 (13)	−0.00061 (16)
Br1	0.0181 (6)	0.0250 (5)	0.0309 (6)	−0.0078 (5)	0.0086 (5)	−0.0009 (5)
Br2	0.0231 (6)	0.0243 (5)	0.0258 (5)	0.0033 (5)	0.0100 (5)	−0.0038 (5)
Br3	0.0224 (6)	0.0243 (5)	0.0216 (5)	0.0040 (5)	0.0022 (5)	0.0029 (4)
Br4	0.0193 (5)	0.0292 (5)	0.0224 (5)	−0.0006 (5)	0.0028 (5)	0.0071 (5)
N1	0.007 (4)	0.015 (4)	0.022 (4)	−0.002 (3)	−0.004 (4)	0.001 (3)
N2	0.016 (5)	0.017 (4)	0.018 (4)	−0.003 (3)	0.011 (4)	−0.004 (3)
C1	0.015 (5)	0.018 (5)	0.021 (5)	0.000 (4)	0.006 (5)	−0.002 (4)
C2	0.024 (6)	0.018 (5)	0.039 (7)	−0.009 (5)	−0.001 (6)	0.001 (5)
C3	0.010 (5)	0.021 (5)	0.037 (7)	−0.005 (5)	−0.005 (5)	0.002 (6)
C4	0.017 (6)	0.019 (5)	0.041 (7)	−0.002 (4)	0.014 (5)	0.002 (5)
C5	0.010 (5)	0.020 (5)	0.022 (5)	0.000 (4)	0.001 (4)	−0.006 (4)
C6	0.014 (5)	0.025 (6)	0.019 (5)	0.005 (4)	0.004 (5)	0.006 (4)
C7	0.022 (6)	0.027 (6)	0.025 (6)	0.002 (5)	0.010 (5)	0.002 (4)
C8	0.027 (6)	0.025 (5)	0.017 (5)	0.010 (5)	0.007 (5)	−0.003 (4)
C9	0.030 (6)	0.028 (6)	0.025 (6)	−0.004 (5)	0.012 (5)	−0.009 (5)
C10	0.018 (6)	0.018 (5)	0.025 (5)	−0.009 (4)	0.001 (5)	−0.010 (4)

### Geometric parameters (Å, °)

**Table:**

Pt1—N2	2.046 (7)	C3—C4	1.386 (14)
Pt1—N1	2.048 (7)	C3—H3	0.9500
Pt1—Br1	2.4412 (10)	C4—C5	1.370 (13)
Pt1—Br2	2.4442 (10)	C4—H4	0.9500
Pt1—Br4	2.4595 (11)	C5—C6	1.443 (14)
Pt1—Br3	2.4756 (11)	C6—C7	1.401 (13)
N1—C1	1.336 (11)	C7—C8	1.391 (14)
N1—C5	1.385 (11)	C7—H7	0.9500
N2—C10	1.354 (11)	C8—C9	1.380 (14)
N2—C6	1.382 (12)	C8—H8	0.9500
C1—C2	1.387 (14)	C9—C10	1.379 (13)
C1—H1	0.9500	C9—H9	0.9500
C2—C3	1.380 (17)	C10—H10	0.9500
C2—H2	0.9500		
			
N2—Pt1—N1	80.6 (3)	C1—C2—H2	120.9
N2—Pt1—Br1	95.5 (2)	C2—C3—C4	120.7 (11)
N1—Pt1—Br1	176.0 (2)	C2—C3—H3	119.7
N2—Pt1—Br2	176.0 (2)	C4—C3—H3	119.7
N1—Pt1—Br2	95.4 (2)	C5—C4—C3	119.0 (10)
Br1—Pt1—Br2	88.50 (4)	C5—C4—H4	120.5
N2—Pt1—Br4	89.1 (2)	C3—C4—H4	120.5
N1—Pt1—Br4	89.5 (2)	C4—C5—N1	120.6 (9)
Br1—Pt1—Br4	89.80 (4)	C4—C5—C6	123.1 (9)
Br2—Pt1—Br4	90.91 (4)	N1—C5—C6	116.3 (8)
N2—Pt1—Br3	89.5 (2)	N2—C6—C7	119.6 (9)
N1—Pt1—Br3	88.8 (2)	N2—C6—C5	115.4 (8)
Br1—Pt1—Br3	91.88 (4)	C7—C6—C5	125.0 (9)
Br2—Pt1—Br3	90.34 (4)	C8—C7—C6	119.2 (10)
Br4—Pt1—Br3	177.93 (4)	C8—C7—H7	120.4
C1—N1—C5	119.7 (8)	C6—C7—H7	120.4
C1—N1—Pt1	126.7 (6)	C9—C8—C7	119.9 (9)
C5—N1—Pt1	113.5 (6)	C9—C8—H8	120.0
C10—N2—C6	120.5 (8)	C7—C8—H8	120.0
C10—N2—Pt1	125.3 (6)	C10—C9—C8	119.9 (9)
C6—N2—Pt1	114.2 (6)	C10—C9—H9	120.0
N1—C1—C2	121.7 (9)	C8—C9—H9	120.0
N1—C1—H1	119.1	N2—C10—C9	120.8 (9)
C2—C1—H1	119.1	N2—C10—H10	119.6
C3—C2—C1	118.3 (10)	C9—C10—H10	119.6
C3—C2—H2	120.9		
			
N2—Pt1—N1—C1	178.8 (8)	C3—C4—C5—N1	−0.4 (16)
Br2—Pt1—N1—C1	−1.2 (8)	C3—C4—C5—C6	−179.3 (10)
Br4—Pt1—N1—C1	89.6 (8)	C1—N1—C5—C4	1.1 (14)
Br3—Pt1—N1—C1	−91.5 (8)	Pt1—N1—C5—C4	179.3 (8)
N2—Pt1—N1—C5	0.8 (6)	C1—N1—C5—C6	−179.9 (9)
Br2—Pt1—N1—C5	−179.3 (6)	Pt1—N1—C5—C6	−1.7 (11)
Br4—Pt1—N1—C5	−88.4 (6)	C10—N2—C6—C7	−0.9 (14)
Br3—Pt1—N1—C5	90.5 (6)	Pt1—N2—C6—C7	178.4 (7)
N1—Pt1—N2—C10	179.6 (9)	C10—N2—C6—C5	179.4 (9)
Br1—Pt1—N2—C10	−1.1 (9)	Pt1—N2—C6—C5	−1.3 (11)
Br4—Pt1—N2—C10	−90.8 (8)	C4—C5—C6—N2	−179.1 (9)
Br3—Pt1—N2—C10	90.7 (8)	N1—C5—C6—N2	2.0 (13)
N1—Pt1—N2—C6	0.3 (6)	C4—C5—C6—C7	1.3 (16)
Br1—Pt1—N2—C6	179.6 (6)	N1—C5—C6—C7	−177.6 (9)
Br4—Pt1—N2—C6	89.9 (6)	N2—C6—C7—C8	0.7 (14)
Br3—Pt1—N2—C6	−88.6 (6)	C5—C6—C7—C8	−179.7 (10)
C5—N1—C1—C2	−1.8 (14)	C6—C7—C8—C9	0.4 (15)
Pt1—N1—C1—C2	−179.7 (7)	C7—C8—C9—C10	−1.3 (16)
N1—C1—C2—C3	1.6 (16)	C6—N2—C10—C9	0.1 (15)
C1—C2—C3—C4	−0.9 (17)	Pt1—N2—C10—C9	−179.2 (8)
C2—C3—C4—C5	0.3 (17)	C8—C9—C10—N2	1.0 (16)

### Hydrogen-bond geometry (Å, °)

**Table:**

*D*—H···*A*	*D*—H	H···*A*	*D*···*A*	*D*—H···*A*
C1—H1···Br2	0.95	2.73	3.366 (9)	125
C3—H3···Br1^i^	0.95	2.89	3.734 (10)	149
C10—H10···Br1	0.95	2.70	3.335 (9)	125

Symmetry codes: (i) *x*+1, *y*+1, *z*.
